# Advanced Carbon Reinforced Concrete Technologies for Façade Elements of Nearly Zero-Energy Buildings

**DOI:** 10.3390/ma15041619

**Published:** 2022-02-21

**Authors:** Robert Kraft, Alexander Kahnt, Otto Grauer, Mike Thieme, Daniel Sebastian Wolz, Dominik Schlüter, Matthias Tietze, Manfred Curbach, Klaus Holschemacher, Hubert Jäger, Robert Böhm

**Affiliations:** 1 Faculty of Civil Engineering, Structural Concrete Institute, Leipzig University of Applied Sciences, PF 30 11 66, 04251 Leipzig, Germany; robert.kraft@htwk-leipzig.de (R.K.); alexander.kahnt@htwk-leipzig.de (A.K.); otto.grauer@htwk-leipzig.de (O.G.); matthias.tietze@tu-dresden.de (M.T.); klaus.holschemacher@htwk-leipzig.de (K.H.); 2 Faculty of Engineering, Leipzig University of Applied Sciences, PF 30 11 66, 04251 Leipzig, Germany; 3 Institute of Lightweight Engineering and Polymer Technology, TU Dresden, 01307 Dresden, Germany; mike.thieme@tu-dresden.de (M.T.); daniel_sebastian.wolz@tu-dresden.de (D.S.W.); hubert.jaeger@tu-dresden.de (H.J.); 4 Research Center Carbon Fibers Saxony (RCCF), TU Dresden, 01307 Dresden, Germany; 5 Institute of Concrete Structures, TU Dresden, 01062 Dresden, Germany; dominik.schlueter@tu-dresden.de (D.S.); manfred.curbach@tu-dresden.de (M.C.)

**Keywords:** concrete, carbon fibres, carbon reinforced concrete, nZEB, resource efficiency, manufacturing

## Abstract

The building sector accounts for approx. 40% of total energy consumption and approx. 36% of all greenhouse gas emissions in Europe. As the EU climate targets for 2030 call for a reduction of greenhouse gas emissions by more than half compared to the emissions of 1990 and also aim for climate neutrality by 2050, there is an urgent need to achieve a significant decrease in the energy use in buildings towards Nearly Zero-Energy Buildings (nZEBs). As the energy footprint of buildings includes the energy and greenhouse gas consumption both in the construction phase and during service life, nZEB solutions have to provide energy-efficient and less carbon-intensive building materials, specific thermal insulation solutions, and a corresponding design of the nZEB. Carbon reinforced concrete (CRC) materials have proven to be excellent candidate materials for concrete-based nZEBs since they are characterized by a significantly lower CO_2_ consumption during component production and much a longer lifecycle. The corresponding CRC technology has been successively implemented in the last two decades and first pure CRC-based buildings are currently being built. This article presents a novel material system that combines CRC technology and suitable multifunctional insulation materials as a sandwich system in order to meet future nZEB requirements. Because of its importance for the life cycle stage of production, cost-efficient carbon fibers (CF) from renewable resources like lignin are used as reinforcing material, and reinforcement systems based on such CF are developed. Cutting edge approaches to produce ultra-thin lightweight CF reinforced concrete panels are discussed with regard to their nZEB relevance. For the life cycle stage of the utilization phase, the thermal insulation properties of core materials are optimized. In this context, novel sandwich composites with thin CRC layers and a cellular lightweight concrete core are proposed as a promising solution for façade elements as the sandwich core can additionally be combined with an aerogel-based insulation. The concepts to realize such sandwich façade elements will be described here along with a fully automated manufacturing process to produce such structures. The findings of this study provide clear evidence on the promising capabilities of the CRC technology for nZEBs on the one hand and on the necessity for further research on optimizing the energy footprint of CRC-based structural elements on the other hand.

## 1. Introduction

The European Green Deal defines the goal of the European Union being the first climate-neutral continent by 2050. The European Climate Act establishes the EU’s voluntary commitment and the first milestone for reaching this goal. The greenhouse gas emissions should be reduced by at least 55% until 2030 compared to 1990. Since then, greenhouse gas emissions have already decreased by 24% while the economy has grown by 60% [1,2].

This indicates a complete reorientation of the building industry towards sustainable building concepts. It is necessary that climate-friendly and resource-efficient arrangements are made regarding building materials and construction levels. Thereby, technical properties of buildings and the high requirements for their service life should be sustainable for future generations. According to the recast of the directive on energy performance of buildings (EPBD), all new buildings should achieve near-zero energy consumption after 2020, meaning the directive supports the improvement of the energy performance of buildings in the EU while taking into account the respective external climatic and local conditions as well as indoor climate requirements and cost efficiency [3]. Hence, there is a high demand for a profound market transformation concerning efficient materials and technologies in the building industry to support the actual implementation of near-zero energy and emission buildings with high indoor air quality throughout in the EU. While the energy consumption of buildings strongly depends on the climate and local weather conditions, other aspects result from the selection of appropriate materials and technical components for a successful implementation of nZEB envelopes. Therefore, innovative building materials are needed in terms of performance, durability, and gray energy. This applies to both new buildings and redevelopment projects.

The most important part of a building from an energy conservation point of view is the building envelope. One way to reduce energy consumption and thus increase the energy efficiency is to improve the thermal insulation. Structural thermal insulation fulfills several tasks today: in addition to the well-known requirements for minimum thermal insulation to ensure condensation and mold-free building component surfaces, it should also provide comfort and a pleasant indoor climate at all times of the year. From an energy perspective, however, the most important requirement is the reduction of transmission heat loss, making structural thermal insulation an important environmental protection issue. The combustion of fossil fuels contributes to the greenhouse effect through emissions from heating among other things.

One approach to optimize concrete constructions with regard to their nZEB potential is to use new high-performance materials and to integrate additional functionalities. Because the Global Warming Potential (GWP) of concrete structures directly depends on the component thicknesses, the scientific problem for material scientists is to provide materials that enable thin lightweight components to be manufactured, preferably entirely from inorganic constituents in order to recycle them easily. Those measures would significantly decrease the gray energy of concrete-based buildings.

Carbon reinforced concrete (CRC) as a composite material made of high-performance fine-grained concrete and a reinforcement of carbon rods or mesh-like CF textiles is the most promising material system for the energy-saving purposes mentioned above. After 20 years of research on carbon reinforced concrete in the German research centres Dresden and Aachen, both the production processes and construction/dimensioning methods were further evolved within the joint research project “C³—Carbon Concrete Composite” [4], which started in 2014. Within the framework of this project, a building has been constructed on the campus of Technical University Dresden using exclusively non-metallic (predominantly carbon) reinforcement [5]. The building consists of two complexes, a double-curved concrete shell—the TWIST element—and the semi-precast BOX complex. The BOX consists of semi-precast wall elements and precast ceiling elements. The TWIST is a light roof-wall shell produced on-site. Frenzel et al. provide detailed information about the design, production, and assembling processes [6]. Further collaborative research on carbon reinforced concrete structures is currently being carried out in a Transregio project (TRR 280), focussing on further structural optimization [7].

CRC is a non-corrosive material. Its durability can lead to a longer service live of building structures. The protective concrete layer—normally required to protect the steel reinforcement from corrosion—can be reduced to several millimetres, resulting in thinner building parts and a reduction in the amount of concrete used [8,9]. For example, the thickness of a steel reinforced concrete façade of 8–10 cm can be reduced to 3 cm thickness [10]. This makes CRC a promising technology to decrease greenhouse gas emissions during the building phase. The environmental or economic sustainability of CRC was therefore analysed in different studies. Various reinforcement technologies for façade elements were investigated by Williams Portal et al. [11], showing that CRC yields to the lowest total environmental impact compared by means of cradle-to-gate life cycle assessment. Laiblova et al. [12] studied an experimental façade with the same conclusion that the average environmental impact of CRC is lower than of steel-reinforced concrete facades. Stoiber et al. [13] compared bridges made of different materials, showing a lower global warming potential of CRC compared to steel-reinforced concrete. Scope et al. firstly conducted a holistic assessment of design variants of sandwich wall systems including ecologic, economic, and social impacts [14]. Within this study, four variants of sandwich wall systems (two of steel reinforced concrete and two of CRC) with similar sound and thermal insulation were compared. The weight of the concrete fraction (−16 to −33%) was significantly reduced using CRC, and the reinforcement material was about 16 to 22 times lower in weight. Subsequently, the two variants of CRC showed a better environmental performance with lower GWP scores, supporting findings of Stoiber et al. However, for the overall (cradle-to-grave) perspective, no clear advantage of CRC could be identified concerning GWP, as underdeveloped end-of-live processes of CRC have a major environmental impact. The development of recycling technologies and how it is already addressed by existing research projects [15] may make up this disadvantage in the future. Currently, these processes are still far from ideal from an environmental perspective as they correspond to downcycling and are associated with high energy consumption for the pyrolysis process. Consequently, the development of an appropriate reuse-and-recycling concept is critical for an environmental impact. On a constructive level, this includes a concept for disassembling material level, on building part level and on building level. Looking at a single CRC sandwich element, Scope et al. indicate that the environmental impact is dominated by the manufacturing stages of concrete and of the CF reinforcement. Insulation material plays a minor role. Within the manufacturing process of the CF reinforcement, the precursor production is the greatest driver. The substitution of petroleum-based poly-acrylonitrile (PAN) may be a convenient measure to decrease overall GWP. Promising alternatives are lignin and cellulose-based PAN [16,17,18]. Regarding the concrete manufacturing process, cement is the main driver, followed by admixtures and aggregates. There are numerous research and development efforts for a low-carbon transition path in the concrete and cement industry [19,20]. An efficient use of the materials is an important approach to reduce material consumption and thus lower the environmental impact. This includes efficient use of the reinforcement through adapted reinforcement guidance (e.g., variable axial reinforcement guidance) and efficient use of concrete through structural optimization. The functionalization of carbon slabs is a further promising approach to reduce material usage [21].

The use of carbon fibres has become established in many industries due to its good properties and potential for sustainability. In the construction industry, attempts have been made for some years to exploit the advantages for ecological and economical construction. Based on previous experience with the use of glass fibre (GF) reinforcements, the focus is now increasingly shifting towards CF, which enables an even greater increase in properties. In addition, due to the increasing demand for CF, further production possibilities need to be developed. The production of “green” fibres based on renewable raw materials such as lignin is therefore a new way to open up the market for the construction industry. This will provide a sustainable and at the same time cost-effective fibre alternative [22,23,24].

Moreover, a consistent use of reinforcement in the construction industry requires a continuous reinforcement system consisting of one reinforcement material. Therefore, in addition to the already established textile reinforcement structures, bar-shaped structures are also necessary to withstand high stresses and provide long service lives. Here, the economic availability and production as well as the utilization of the excellent properties of CF and new functionalizations are the focus of current research and development [25,26].

Thermal insulation materials are an essential component for reducing the energy consumption and energy costs of buildings and thus are an indispensable part towards the energy transition. However, they also offer many other important functions for the user and for maintaining the value of the building. Sufficient thermal insulation is a prerequisite for a hygienic and comfortable indoor climate while protecting the building components from moisture and frost damage [27]. When choosing a suitable insulating material, a good insulating performance for the reduction of thermal conductivity is not the only issue to consider. Other important properties include density, dimensional stability, compressive strength, health and safety aspects, as well as flammability. Cost efficiency depends on the price of the insulation material and its installation, but also on durability, service life, and energy balance. The ecological evaluation mainly depends on the energy and CO_2_ balance, the environmental compatibility, and the possibilities of material recycling or disposal.

With increasing requirements for energy efficiency in buildings and the growing shortage of space in urban areas, high-performance insulation materials have emerged in recent decades. The range of application has expanded, and new processing techniques have been developed. Among them are high-performance insulation materials to improve thermal conductivities such as vacuum insulation panels (VIP) [28] or aerogels [29,30]. However, with the increasing shortage of fossil materials and the growing sustainable awareness, substitutes need be found for market-dominating fossil insulation materials such as expanded polystyrene (EPS) or extruded polystyrene (XPS). The production and use of aerogel foamed concrete in prefabricated sandwich panels is a good opportunity to replace those commonly used fossil materials. Various studies [31,32] have shown that by using aerogel foamed concrete, non-combustible, highly efficient, and well recyclable building elements can be produced from purely mineral materials. However, the current state of research does not reach beyond a laboratory scale and individual demonstrators. In order to be able to produce aerogel foam concretes cost-efficiently and thus be competitive on the insulation market, the manufacturing processes have to be rethought and transferred to an automated production as best as possible.

From the results of the current studies on sustainability of the concrete-based structures mentioned above, several fields of action can be derived for further improvement of materials for wall systems, including (i) pure material substitution (e.g., lignin for CF), (ii) material reduction using structural optimization, and (iii) solutions for deconstruction, reuse and recycling at the building level, component level, and material level. Nevertheless, these technologies can only be widely deployed if they can compete economically with existing products. This leads to another field of action, (iv) the development of a viable cost-efficient production process. Within this paper, the authors present a novel material sandwich-like system based on the previously mentioned field of actions. The novel façade elements contain high-performance materials both for structural and insulation purposes that have been chosen with regard to their suitability to reduce gray energy. In particular, material reduction using material substitution (fibre level and insulation level) and structural optimization of the reinforcement geometry will be examined in detail along with the development of an associated manufacturing process for the automated and resource-efficient mass production of nZEB façade elements. A best practice material solution that combines state-of-the-art high-performance materials using semi-automated manufacturing processes will be shown along with open scientific questions to achieve a commercial breakthrough of these new materials. The consequent use of those high-performance materials with low-cost raw materials will result in lightweight, adaptable, and material-efficient building components.

## 2. Carbon Reinforced Concrete Composites

### 2.1. Materials

#### 2.1.1. Low-Cost Carbon Fibres

CF are produced by a thermomechanical process chain consisting of a stabilization, a carbonization, and a possible graphitization step. To date, most commercial CF are based on petroleum-based polyacrylonitrile and are produced in conventionally heated furnaces [25]. While the production costs are estimated to be ~9 to 30 €/kg in terms of tow size [33,34], mass production reduces the CF cost problem. Nevertheless, the conventional process steps lead to high CO_2_ emissions due to the advanced polymerization processes, natural gas or electrically heated furnaces, and waste treatments [35]. Therefore, new technologies such as vacuum-assisted stabilization processes [36] or switching to renewable precursor fibre compositions of lignin, cellulose, and renewable PAN [26,36,37,38] aim to minimize or eliminate the CO_2_ emissions generated. In addition, the use of renewable feedstocks as polymeric precursors for CF would broaden the potential CF production base, as commercially available PAN sources are limited.

**Figure 1:**
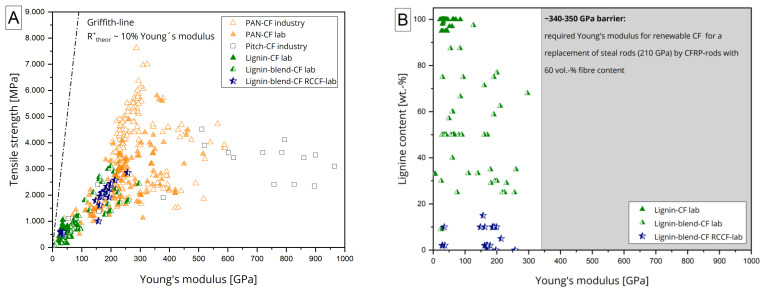
Overview of the mechanical properties of PAN-based, pitch-based, lignine-based, and lignine-blend-based carbon fibres with filament diameters above 3 µm (**A**) and in case of lignin-based CF replacing steel rods with lignin-resin based CF-reinforced rods (**B**).

A brief overview of CF properties and compositions published to date is provided in Figure 1A. While PAN- and pitch-based CF are already in commercial use, renewable CF are not yet commercially available as they are the subject of ongoing research projects. In addition, there are some industrial development activities [39,40]; the parameters shown for lignin- or lignin-blend-based CF are mostly based on batch processed fibres. Upscaling to continuous processes with published lignin or lignin blend CF greater than >50 GPa has not yet been achieved [41].

Therefore, continuous CF with at least partial replacement with lignin has not yet been developed. In order to replace conventional steel reinforcing bars with a Young’s modulus of 210 GPa (DIN 488-2), the CF-based rods must have at least the same Young’s modulus to get accepted by end-users. Assuming the classical rule of mixture for unidirectional composites.

(1) $$E_{rod}=E_{f}\cdot v_{f}+E_{m}\cdot \left(1-v_{f}\right)$$

to be valid, a desirable fiber volume content *ν_f_* of 60% and a polymer Young’s modulus of approx. 7.5 GPa (typical value for the resins currently used), the CF would need to have a Young’s modulus of 340–350 GPa to meet this requirement. According to the state of the art, these values are not yet achieved; see Figure 1B. To allow lower requirements for renewable CF, building codes would need to be changed, but still the CF tensile modulus would need to be in the range of >250 GPa, which only a few CF in research today meet [33,34]. Therefore, in order to develop renewable lignin-based CF, much research needs to be done. This includes the investigation of lignin and lignin-blend compositions, specifically adapted and further developed spinning systems for multicomposition precursors, and even improved thermal conversion strategies.

#### 2.1.2. CF-Based Reinforcement Systems

**Figure 2:**
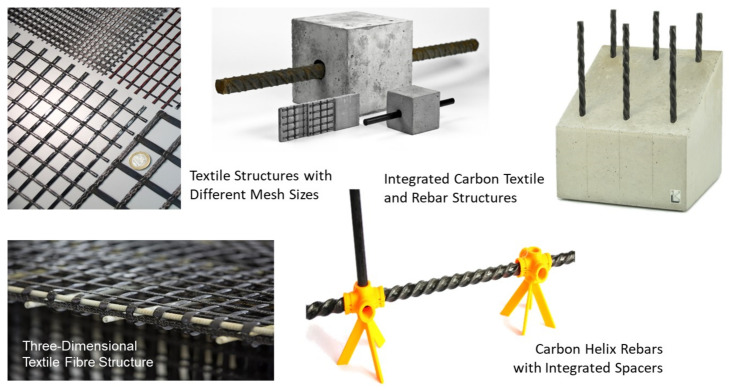
Novel carbon fibre reinforcement systems: textile preforms and bar-shaped rods.

In addition to raw materials, the used reinforcement systems and their manufacturing processes also have a major impact on sustainability and eco-balance. As Figure 2 indicates, there are generally two types of CF-based reinforcements: planar CF textile preforms and bar-shaped reinforcement rods.

**Figure 3:**
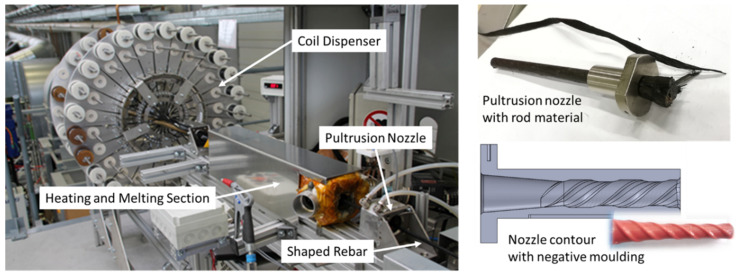
Helix-pultrusion and design of the nozzle geometry for profiling.

The optimal utilization of the high-performance potential of the CF reinforcement is very important for those reinforcement systems. Since the close-meshed textiles transfer the rather low fibre forces very uniformly into the concrete due to the mesh formation and the nodal points, this two-dimensional reinforcement system is currently already widely used in applications. In contrast, bar-shaped reinforcing rods are currently still a very new reinforcement since the transfer of the very high rebar forces to a very concentrated area in the concrete cross-section is still a major challenge. This requires an adapted design of the rebar profile. To optimize the load transfer, the aim is to achieve an optimum between a pronounced outer profile geometry for a good bond to the concrete, a straight orientation of the carbon fibres in the rebar cross-section for good rebar tensile values, and an economical and sustainable production of the rebars. Based on extensive investigations, the so-called helix pultrusion has proven to be an effective way to achieve this optimum for the CF rebars [26]; see Figure 3.

In particular, the simple manufacturing process in just one production step represents an economical and resource-saving solution. Compared to the additive processes commonly used with several production steps for bar forming and a subsequent profiling, a direct profiling is possible here during the manufacturing process. This also means that there are no further process steps that require additional energy or resources. Additionally, compared to the so-called subtractive processes, in which the expensive CF material has to be cut off the initial cross-section in a timely and energy-consuming process, helix pultrusion is a sustainable process with no waste products.

**Figure 4:**
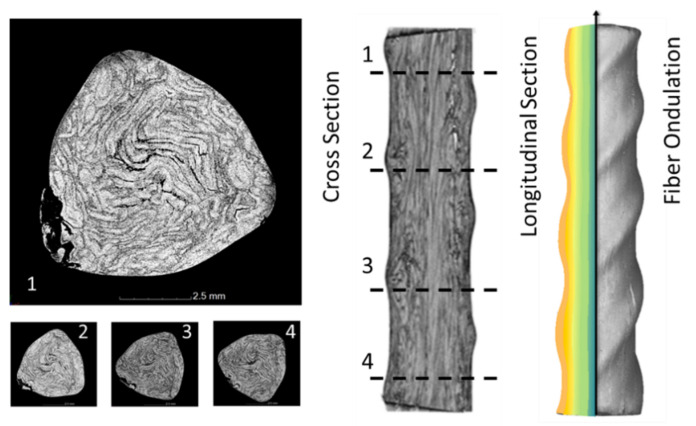
Computed tomography pictures to evaluate the axial and transverse fibre orientation in helix rebars.

In order to evaluate the relationship between the inner fibre layer and the outer profile geometry, parameter studies were carried out to optimize the rebar geometry based on optical scanning measurements (ATOS SO 4M from GOM GmbH, Brunswick, Germany) and computer tomography analyses (nanotom 180 NF from Phoenix|X-ray Systems & Services GmbH, Wunstorf, Germany). Based on continuous cross-sectional CT images of the rebars (see Figure 4), the position of the carbon fibres in the longitudinal and transverse direction of the rebars can be empirically described. By combining this information with the measured external rebar geometry, a process model to optimize the pultrusion nozzle and the nozzle contour (see Figure 3) can be derived taking the local strength of the reinforcement into account as well.

As a result, material-specific reinforcement systems both for planar and bar-shaped reinforcement structures are available now to reinforce CRC structures. They can be realized both with conventional PAN-based CF and with sustainable, cost-effective “green” fibres. Very thin and sustainable structures with newly integrated functions to improve the life cycle assessment can be realized in this way.

#### 2.1.3. Aerogel-Based Insulation

Aerogels are highly porous solids with pore sizes in the nanometer range. They have only been in use in the building industry for a short time. Due to their special thermal insulation properties, they are currently among the most efficient insulation materials with the lowest thermal conductivities. Mostly, they are based on SiO_2_, which is processed from aqueous precursor solutions containing silicate by adding a base to increase the pH-value [29]. Thus, a highly open-pored, porous SiO_2_ structure is obtained after supercritical drying. A high number of nanopores and a low solid content characterizes this structure. Compared to conventional insulation materials, the pore radius of aerogels is a thousand times smaller, which significantly reduces the heat transfer processes within the material. Thus, the convective part of the heat conduction can be excluded. Pure aerogels usually have a thermal conductivity between 12–20 mW/(m∙K) and the air-filled pores make up to 99.8% of the volume [29]. In addition, SiO_2_ aerogels are nonflammable and have low strengths.

Aerogels are constantly evolving with regard to production process and the final product itself. The challenges in using them for building purposes are the very low densities and the associated high fragility, which makes it difficult to handle without breaking the aerogel products. Therefore, many different composites have been developed to create a more robust material. By combining them with carriers such as polyurethane, the mechanical properties of aerogels are greatly improved.

**Figure 5:**
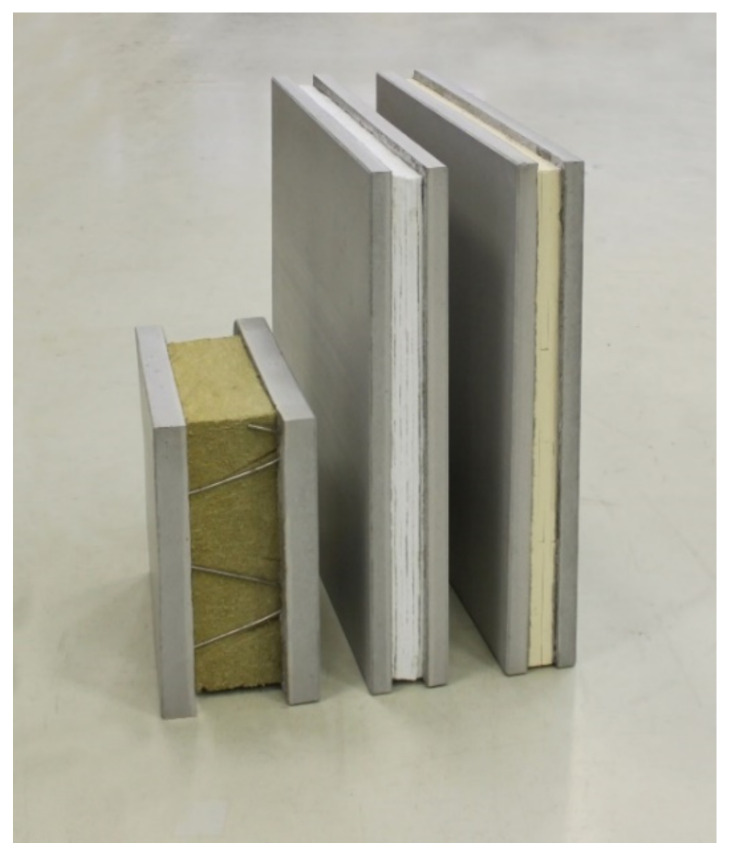
Comparison of a conventional reinforced concrete wall element (**left**) with two CRC wall elements with different aerogel insulation (**middle**, **right**).

Together with BASF (Ludwigshafen, Germany), the high-performance SLENTITE^®^ polyurethane-based insulation panels were examined and characterized here. Studies on the thermal conductivity with a heat flow meter showed a nominal thermal conductivity of 18 mW/(m∙K), resulting in half insulation thicknesses compared to conventional insulation materials. The board dimensions of 550 mm × 370 mm × 15 mm derive from the autoclave drying process during manufacturing. In order to reduce the drying time, the layer thickness was limited to 15 mm. From a mechanical point of view, the plates have a high compressive stress of more than 300 kPa. On the other hand, the disadvantages include high brittleness at higher tensile stresses and low ductility, which allows only a low deformation. High-performance insulating materials such as SLENTITE^®^ are already achieving cost-efficiency in urban areas. Figure 5 shows a wall element made of steel-reinforced concrete with mineral wool (left) compared to two wall elements made of CRC with a SiO_2_ aerogel SLENTEX^®^ (middle) and a polyurethane aerogel SLENTITE^®^ (right). All three wall elements have the same thermal resistance of 3.6 (m²∙K)/W.

### 2.2. CRC Structural Systems

**Figure 6:**
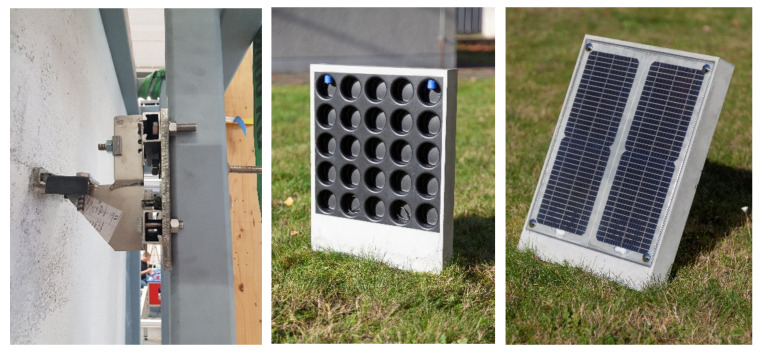
Electrically activatable connection made of shape memory alloy (**left**); CRC panel with integrated photovotaics, voids, and capillary system, taken from [44] (**middle and right**).

The design of façade panels (e.g., sandwich structures) made of CRC needs to be more optimized to further reduce the environmental impact towards higher levels of reuse and recycling (demountable design) and lower material consumption (structural optimization). To ensure a long service life, even if parts of the building need to be repurposed or renovated, the façade system must allow for a high degree of reuse. For adequate recycling at the end of the life cycle, the structure must be disassembled into its individual elements and materials. Therefore, the anchorage between the sandwich panel and the adjacent panels or the supporting structure must be appropriately detachable. In addition, the individual layers of the sandwich panels must be designed in such a way that they can be dismantled without being destroyed. There are existing anchoring systems such as the HALFEN-FPA-5-SL30, which are designed for thin-walled CRC elements. Newly developed concepts are based on shape memory alloy allowing for a blind and non-mechanical assembling [42]. Figure 6 shows such a connector, consisting of a U-anchor-channel and an inserted shape memory element that can be electrically activated. However, they do not yet allow easy disassembly of the panels.

As shown above, the use of a CF-based reinforcement can lead to massive material savings. However, the reduced element thickness leads to a reduced effective height or increased deflection. By embedding voids within the panels, the flexural load capacity can be increased while using less material (see Figure 6). The use of hollow bodies or voids in reinforced concrete structures is state of the art [43], but is not yet used in CRC. Another way to save material is to improve the use of reinforcement material. The high flexibility of the reinforcement allows easy guidance of the reinforcement within the concrete element. Schlueter et al. [44] presented a strategy to combine these different optimization approaches by using a semi-finished product that already contains voids, load-adjusted reinforcement, and anchoring systems.

### 2.3. Environmental Benefit of CRC Technology

Aiming for sustainability-oriented innovation, façade elements must not only reduce energy consumption during the use phase (e.g., through insulation and integrated energy harvesting) but also have a low primary energy required for production. Strategies include material minimization by developing efficient manufacturing methods, new form findings for load-bearing structures, or using new high-performance materials. The shape-flexibility, corrosion resistance and the high strengths of the CF-based reinforcement pays into these strategies. A protective concrete layer—needed to protect steel reinforcement from corrosion—is not required, leading to thin and lightweight structures [8,9]. The shape-flexibility of the reinforcement allowing for a load-oriented and form-flexible design [45] leads to further material reductions. De Brito and Kurda developed strategies to further reduce negative impacts of concrete, mentioning the use of CF-based reinforcement [27].

Although the potential for material savings through CF-based reinforcement is obvious, quantifying the sustainability potential along the entire life cycle is more complex and depends on various aspects like the specific application scenario and functional units, making generally valid statements difficult.

Williams Portal identified four reinforcement material scenarios (alkali resistant glass, basalt fibre, carbon fibre, and steel rebars), finding CRC scores lowest in GWP [11]. Scope presented a life cycle analysis for CRC wall systems also including social and economic assessment [14]. CRC performed best in all dimensions of sustainability compared to steel-reinforced wall systems. González et al. compared carbon fibre reinforced polymers reinforcement to steel reinforcement in a cradle-to-gate Life Cycle Assessment (LCA) [46]. They found that the CF-based reinforcement has about half the embodied energy of the steel reinforcement. They also found that CF reinforcement is advantageous due to its lightness and ease of assembly reducing accidents, its shorter mounting time, and less noise and air pollution.

Garg and Shrivastava, however, showed that glass fibre rebars have lower GWP emissions than basalt or carbon fibres looking at the ultimate moment capacity [47]. Laiblová et al. compared façade panels with different materials and found basalt fibre-reinforced concrete scores lowest in most environmental impact categories examined [12]. These results show that each application scenario needs to be assessed individually to reach the best material choice.

A further aspect of sustainability is material recyclability. Recent studies have shown that carbon fibres can be recovered from carbon reinforced concrete elements using conventional deconstruction technologies [48]. The reuse of such recovered fibres in new CRC products is the subject of ongoing research and development [49].

## 3. Energy-Efficient CRC-Based Façade Elements

### 3.1. Conventional Sandwich Elements

Conventional reinforced concrete sandwich panels were developed in the 1950s. With them, it was quickly possible to create affordable housing. Typical examples are residential buildings from the 1960s and 1970s that can still be found throughout Europe today. The complete factory production made it possible to produce efficient, durable, and high-quality concrete sandwich elements, regardless of the weather. Due to their low assembly effort, they are highly economically. The precast concrete elements consist of three layers: a facing shell, an insulating shell, and a load-bearing shell [50]. These layers fulfill individual and as a whole different tasks. Due to the structure with a lightweight core and comparatively thin outer layers, sandwich elements have good properties, such as high specific load-bearing capacity, high fatigue strength, good sound insulation, and excellent heat insulating properties. So far, sandwich elements have been produced of reinforced concrete with a top layer thickness between 70 and 100 mm. This required thickness derives from the external loads and the maintenance of the concrete coverings. Only for the protection of the steel reinforcement from corrosion, approx. 50% of the material has to be used, although only 3–4 cm are required for the load-bearing capacity. The top layer is usually non-load-bearing and is connected to the load-bearing layer with rust-proof fasteners. The inner shell is often designed as a load-bearing shell with a thickness of approx. 10 cm, which transfers the vertical loads to the supported floor slabs. It is also used for the stiffening load-bearing effect of the sandwich element. In this case, the component thickness is mostly determined by the load-bearing capacity. For the sandwich core, primarily petroleum-based insulating materials such as expanded or extruded polystyrene (EPS, XPS) and polyurethane-based insulating materials (PUR) are used. If there is an increased requirement for fire protection, non-combustible insulating materials made of mineral fibers such as rock wool or glass wool are applied [28].

### 3.2. Sandwich Elements with CRC Layers and Aerogel Insulation Core (Slentite^®^/Slentex^®^)

In contrast to conventional façade elements, various projects showed a significant reduction of the façade thickness and material input, when using CRC panels and high performance insulating materials. However, these components consist of organic and inorganic composite materials [51].

#### 3.2.1. Structure of the Elements

**Figure 7:**
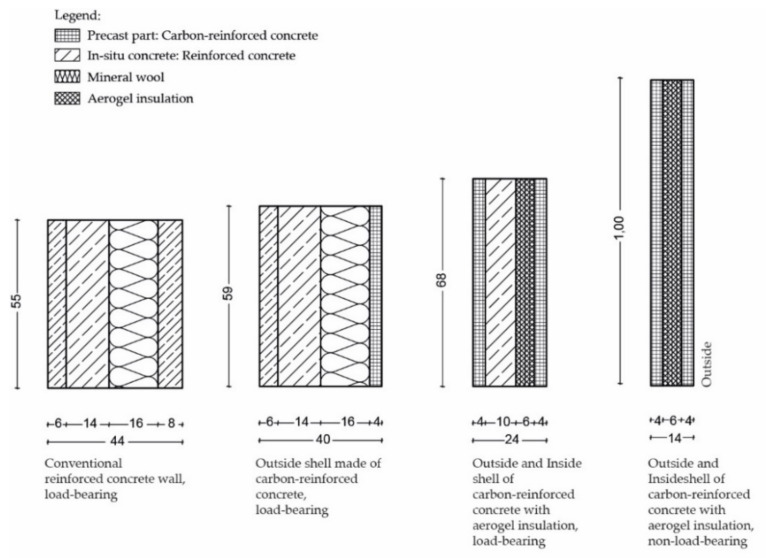
Comparison of different concepts for concrete facades: high-performance materials can significantly reduce wall structures, thus lowering the gray energy in building materials (mass of all elements: 200 kg).

Figure 7 shows the concepts of different load-bearing and non-load-bearing CRC façade elements, including their geometry, compared to conventional reinforced concrete façade elements. These concepts were validated both in research projects and in practical applications [5,6,52]. The aim was to reduce the wall thickness in order to save materials and gain additional usable space in the building. This was achieved by using CRC shells of only 4 cm thickness and aerogel insulation. As a result, the load-bearing wall thickness was almost halved from 44 cm to 24 cm. The façade elements shown in Figure 7 all have a weight of 200 kg. With decreasing thickness by using innovative composite materials, a significantly larger area within the building can be realised.

**Figure 8:**
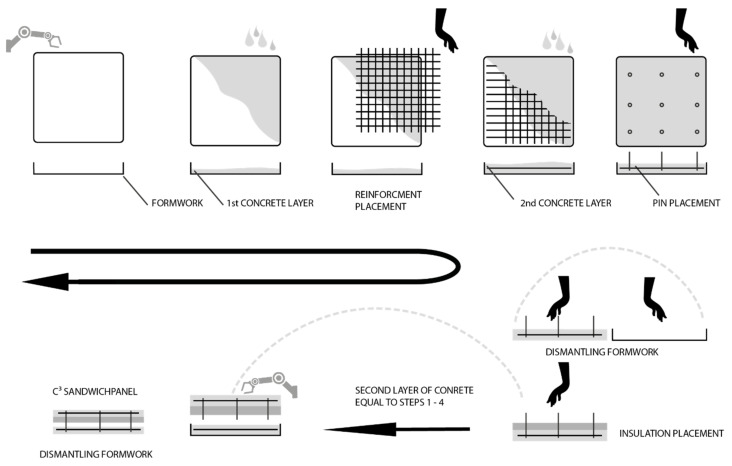
Schematic production of CRC sandwich elements.

Currently, the production of sandwich elements with CRC is only partially automated (see Figure 8). At the beginning, like for conventional reinforced concrete, the formwork is produced by formwork robots. The wall layers are placed in a two-stage concreting process. This means that firstly one layer of concrete is put into the formwork and is then vibrated briefly to achieve a possible self-leveling. Afterwards, the previously manually processed carbon fibre grid is placed by hand on top of the concrete layer and then covered with a second one. Finally, the concrete is vibrated again and the connecting elements (pins) are added. After hardening, the first sandwich component—the outer shell—is stripped from the formwork. The insulation is then placed by hand and at the same time, the inner shell is concreted identical to the first layer without pins. Before the inner shell hardens, the outer shell is placed into the fresh inner shell. Once the hardening process is finished, the two shells are connected to each other by the connecting elements [53].

#### 3.2.2. Handling of Carbon Fibre Grids

The carbon reinforcement currently used for the sandwich elements is purchased as a semi-finished product from external manufacturers; see Section 2.1.2 and Figure 2. They supply the carbon fabrics to the concrete plants in the form of prefabricated mats or rolls. Before the reinforcement can be installed, it has to be cut to the required dimensions. For recesses such as doors or windows, these areas need to be cut out as well. If two mats are positioned next to each other, the statically relevant force transmission must be ensured. The mat sections are therefore placed with a defined overlap. However, the double reinforcement layer in these areas is not required for the design. Thus, unnecessary material is used for the overlap to save material. However, for door and window recesses as well as for protruding reinforcement areas, there may also be unnecessary waste. In very few cases, that can be used for other purposes.

**Figure 9:**
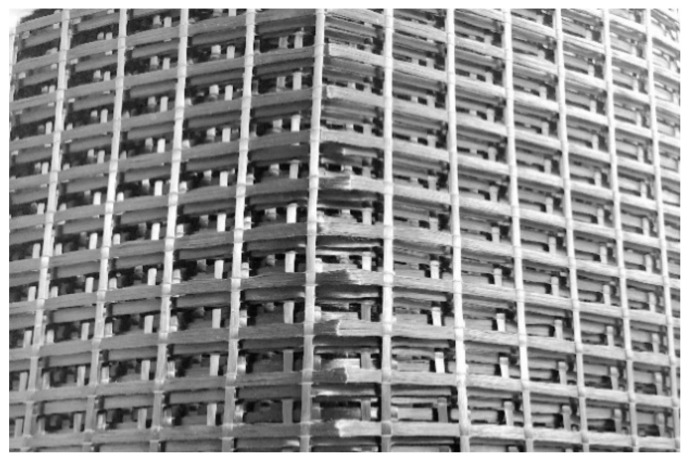
Damage in CF textiles due to transport.

An important step within the textile reinforcement process chain is the transport from the semi-finished product manufacturer of the CF mat to the concrete plant. Improper handling of the mats can expose them to transverse forces that the carbon fibers cannot withstand. Consequences are the (partial) destruction of the continuous fiber, and the force distribution within the CF mat is no longer guaranteed. For example, Figure 9 shows damaged longitudinal fibers of a CF mat, even though the reinforcement was delivered in a coiled carbon mesh. This can occur when several rolls are stored on top of each other, when the lower rolls are compressed and the permissible bending radius is undershot. Such reinforcement can no longer be used. The need to establish sophisticated quality assurance measures in the entire process chain must therefore not be underestimated.

## 4. Improvement of the CRC Technology towards nZEB

Several open scientific questions to upscale and commercialize the CRC technology and to overcome the remaining problems mentioned in the previous chapters will be tackled in the European Horizon 2020 project iClimaBuilt (Functional and advanced insulating and energy harvesting/storage materials across climate adaptive building envelopes). As part this project, a pilot line (see Figure 8) for the production of sustainable cement- and concrete-based sandwich façade elements is further developed and automated, enabling the manufacturing of CRC sandwich elements with additional functions. This chapter introduces the concept for this near-industrial pilot line. The requirement for the fully automated line is to produce an all-mineral, modular, self-supporting sandwich element with optimized thermal material properties (test cases) in a cost-efficient way. The functional design of the sandwich elements must be able to be adjusted to different climatic conditions in Europe. At the end of iClimaBuilt, the sandwich elements will be installed in so-called living labs for long-term testing and monitoring.

### 4.1. Concept: Cellular Lightweight Concrete (CLC) with Aerogels

The test case is a load-bearing sandwich panel for the façade, where the thermal performance of the insulation is to be improved by incorporating silica aerogels into a porous cellular lightweight concrete (CLC) sandwich core. The silica aerogels are based on recent developments published in [54,55]. CLC, also known as foam concrete, is a lightweight cementitious material that consists of cement, sand (optional), water, and foam [55,56]. Its thermal conductivity and price is comparable with EPS. If silica aerogel is added, the thermal conductivity can even reach 30 mW/(m∙K). CLC is non-flammable and does not release any toxic gases when heated up. It presents an affordable and sustainable alternative with both structural and insulating characteristics. Since it consists of 99% of mineral components, it can be easily used as a secondary resource, e.g., as material for cement manufacturing or as filler for concrete production. The technology to produce ultralight CLC structures is based on the works published in [31].

The combination of CLC and aerogels is expected to drastically reduce the thickness of the insulation layer by half while maintaining thermal efficiency. CLC with different densities in the range of 60–120 kg/m² with different dosages of microfibres and aerogels are expected to be the best parameter set. CLC is incorporated into the panels, either during casting or in the form of prefabricated panels or blocks. The thickness of the panel is based on the structural capacity of the element. The panels are designed to perform primarily structural, thermal, hygric, acoustical, and aesthetic functions. The façades are designed to span vertically between two floors with maximum dimensions of 3 × 5 m² and maximum weight of 225 kg/m². Since the elements are load-bearing, the sandwich structures not only carry the acting forces of wind, temperature, and dead loads, but also transfer the imposed load acting on the ceiling via the façade. The inner and outer layers are made of high-strength CRC, with the inner layer being approx. 6 cm and the outer layer 3 cm thick. This concept provides a purely mineral façade element that is slim, automatically produced, and can be recycled as well.

### 4.2. Production

As shown in Section 3.2.1, conventional sandwich elements made of CRC can already be produced on partially automated production lines. The elements, which had mostly been manufactured by hand so far, can thus be produced more cost-effectively and with a consistent quality. However, the CF textile reinforcement still has to be placed manually. Textile reinforcements are currently available in various forms. They are offered as mats rolled up on a sleeve or as cut-to-length sheets in standard widths and lengths. This results in a high level of waste of expensive materials for different component sizes, which has to be disposed of at a great expense. Furthermore, a predefined overlap length has to be maintained for mesh joints in order to guarantee a force-fit bond, which consumes additional material.

**Figure 10:**
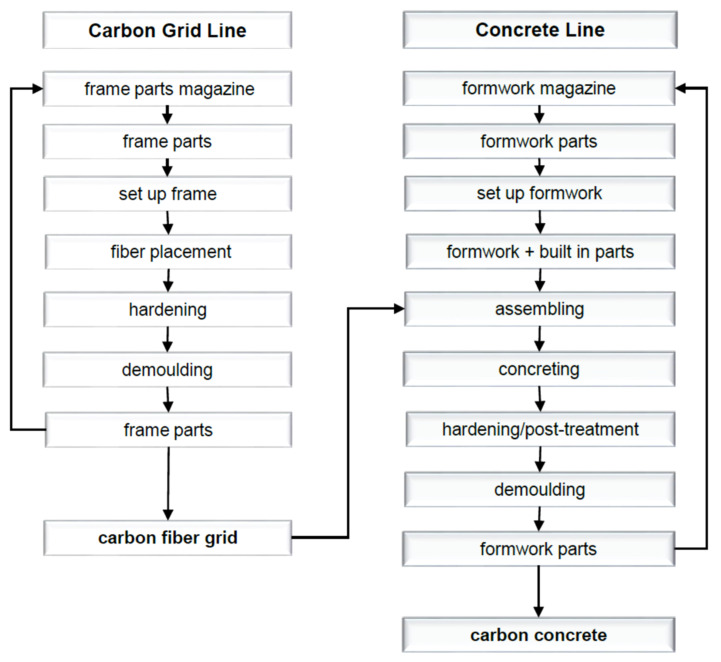
Scheme of the automated pilot line for concrete-based materials.

Automated and load-oriented deposition of the CF rovings enables new wall constructions to be manufactured in a resource-saving and efficient manner. For this purpose, the Carbon Concrete Technology Center was established at Leipzig University of Applied Sciences in 2020, which aims to remove the aforementioned obstacles to the mass production of CRC and thus to pave the way for new markets. The new manufacturing process to produce not only CRC sandwich elements but also other lightweight concrete-based structures is now fully automated and follows the production steps shown in Figure 10.

**Figure 11:**
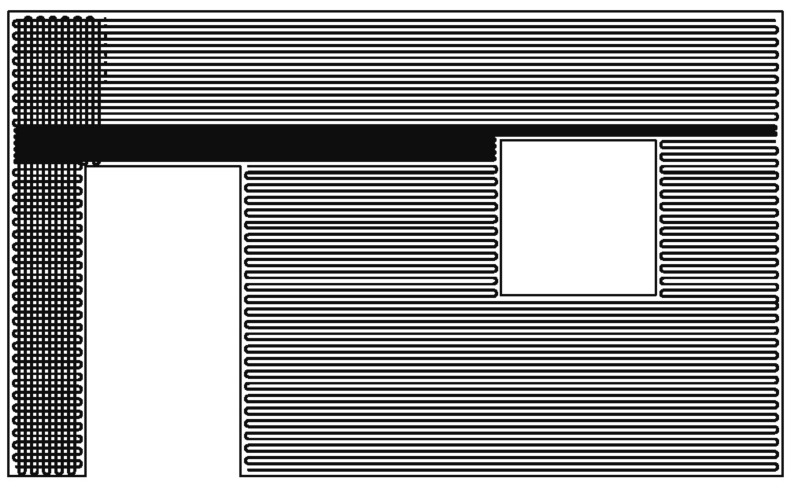
Customized planar CF textile reinforcement for a door-window wall element.

**Figure 12:**
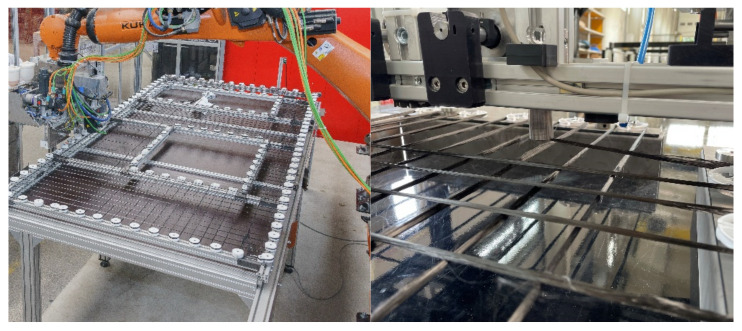
Automated positioning of a freely designed CF textile grid as part of the carbo grid line.

The planar 2D textile reinforcement is produced directly from the CF roving at the application site. For this purpose, the carbon coils are attached to a robot. After an online coating with a resin matrix (Manufacturer: Sika Deutschland GmbH, Bad Urach, Germany), the impregnated CF roving is placed at the desired positions. The main advantage of this process step is that it enables a free design of reinforcement geometries. The reinforcement geometry is digitally transferred to the robot according to the static requirements. The end effector converts the data into a laying path and follows this path. Using this approach, the reinforcement can now be produced individually and load-adapted, e.g., according to a structural optimization (see Figure 11 and Figure 12). Since the CF material usually comes from a continuous roll, this is also a no-waste process. Recesses such as for doors and windows are not part of the laying track, whereas special areas like restrictions of crack width in corners or statically adjusted areas (e.g., lintel) are placed with a tighter mesh. With this zero-cut process, all planar reinforcement geometries can be produced from one piece without any problems.

After the placement, the resulting textile mats are subjected to a temperature process to cure the resin, and the final stiffness of the reinforcement is achieved. With this new process for the CF reinforcement production, material savings of up to 40% were achieved, compared to the conventional production with externally produced semi-finished products in the form of rolls or mats.

## 5. Conclusions

The European directive on energy performance of buildings (EPBD) defines criteria for Nearly Zero Energy buildings (nZEB) that new buildings in Europe should meet after 2020. Since concrete structure are extremely CO_2_ consuming, material scientists in civil engineering have to provide more sustainable solutions for future buildings. The carbon reinforced concrete (CRC) technology is very promising in this context since it drastically reduces the energy and greenhouse gas consumption in the construction phase. However, to develop concrete-based nZEB solutions, specific thermal insulation solutions to decrease the emissions during service life are needed as well. This article identified four different actions to develop the CRC technology further into the nZEB direction:

- substituting conventional raw materials with renewable materials,
- structural optimization,
- deconstruction, reuse and recycling and
- cost-efficient automated manufacturing processes.

Using carbon-reinforced concrete sandwich composites for façade elements as an example, it has been shown that a novel automated manufacturing process allows a cost- and resource-efficient production of such structures. Above all, compared with conventional manufacturing concepts using commercial textile preforms, the presented concept enables a significant material saving potential of approx. 40%. Due to the direct load-adapted deposition, almost no waste is produced within this process. In order to validate the carbon fibre placement and to further develop the manufacturing process, a test case was successfully performed on the new pilot line using conventional carbon fibre rovings, which are directly placed and coated. The sustainability of this process can be further enhanced when the conventional fibres are replaced with carbon fibres from renewable resources like lignin. However, the mechanical properties of such fibres and subsequently their manufacturing processes have to be improved for that purpose. It has been demonstrated that these lignin-based CF can also be used to produce CF-based rebars using a novel manufacturing process called helix pultrusion. After direct placement of the reinforcement, a high-strength concrete mix is produced with the aim of further reducing the clinker content and thus further lowering the gray energy of the building material.

It has been shown that insulation properties can be significantly improved when sandwich panels are produced when a mineral foam sandwich core with aerogels is to be integrated instead of conventional thermal insulation materials. However, the study also revealed that it is necessary to work towards automated manufacturing processes for such high-performance building materials in order to stay competitive. Future work (within the EU project iClimaBuilt) therefore has to include the optimization of the pilot line, material models for all relevant materials, and an experimental validation of the mechanical and thermal properties in different climate zones, together with energy and greenhouse gas consumption, as so-called test cases for nZEB envelopes. Because all these materials are currently developed either on a laboratory scale or on a pilot scale, there is still great potential for environmental improvements that are expected to happen during upscaling processes.

## Data Availability

Not applicable.
